# Hypermetabolic Thyroid Incidentaloma on Positron Emission Tomography: Review of Laboratory, Radiologic, and Pathologic Characteristics

**DOI:** 10.1155/2017/7176934

**Published:** 2017-08-20

**Authors:** Mehrdad Bakhshayesh Karam, Abtin Doroudinia, Farzaneh Joukar, Kobra Nadi, Atosa Dorudinia, Payam Mehrian, Abbas Yousefikoma

**Affiliations:** ^1^Chronic Respiratory Diseases Research Center, National Research Institute of Tuberculosis and Lung Diseases (NRITLD), Shahid Beheshti University of Medical Sciences, Tehran, Iran; ^2^Tracheal Diseases Research Center (TDRC), National Research Institute of Tuberculosis and Lung Diseases (NRITLD), Shahid Beheshti University of Medical Sciences, Tehran, Iran

## Abstract

**Introduction:**

Incidental hypermetabolic thyroid lesions on Positron Emission Tomography have significant clinical value and may harbor malignancy. In this study we evaluated laboratory, radiologic, and pathologic characteristics of incidental hypermetabolic thyroid lesions.

**Materials and Methods:**

We evaluated 18 patients prospectively with various malignancies and hypermetabolic thyroid incidentaloma. The thyroid function tests, ultrasound assessment, and guided FNA biopsy were performed on all cases.

**Results:**

We included 9 male and 9 female patients with mean age of 51 years. Most common malignancy was colon cancer. Metabolic activity quantification using maximum standard uptake value demonstrated range between 1.4 and 65.4 with mean value of 9.4. We found highest metabolic activity in patients with lung adenocarcinoma, B-cell lymphoma, and colon adenocarcinoma. On ultrasound exam most thyroid lesions were of solid, hypoechoic, noncalcified nature with either normal or peripheral increased vascularity. FNA biopsy report was benign in 15 cases and malignant or highly suggestive for malignancy in 3 other cases. Two of the three malignant cases demonstrated metabolic activity higher than average SUV max.

**Conclusion:**

Most thyroid hypermetabolic incidentalomas are benign lesions, while higher values of SUV max are in favor of malignancy. This mandates further evaluation of incidentally found thyroid hypermetabolic lesions on routine PET/CT scans.

## 1. Introduction

Whole body Positron Emission Tomography/Computed Tomography (PET/CT) scan using 18F-Fluorodeoxyglucose (FDG) which is an indicator of cellular metabolic activity is increasingly utilized in initial workup and also follow-up of various medical conditions including malignancies. Occasionally increased FDG uptake may be found in thyroid gland when PET/CT is performed for evaluation of an unrelated medical condition. This finding is referred to as “FDG-avid or hypermetabolic thyroid incidentaloma.”

Thyroid incidentaloma is usually found in asymptomatic patients and although they are not commonly palpable, they may be discovered during evaluation of the neck for other medical conditions. In pathologic assessment, the overall rate of malignancy in thyroid incidentaloma is low, between 5% and 10%, and type of malignancies usually includes papillary or follicular variant of papillary thyroid carcinoma [1].

Detection of thyroid incidentaloma is usually performed through cervical ultrasound examination (US), Computed Tomography (CT), Magnetic Resonance Imaging (MRI), and 18F-Fluorodeoxyglucose Positron Emission Tomography (FDG PET).

FDG PET/CT has been in clinical practice for staging and follow-up of cancer patients with various organ system involvement. The basis of FDG PET/CT is detection of areas with increased level of metabolic activity. Increased metabolic activity requires more glucose consumption by the affected tissue and because 18F-FDG is radio-labeled analog of glucose, more uptake of 18F-FDG is expected in the tissues with increased rate of metabolic activity which are called “FDG-avid” tissues [2].

FDG-avid thyroid incidentalomas are described as focus or foci with increased metabolic activity within the thyroid gland [2]. The incidence rate of FDG-avid thyroid incidentaloma differs between 0.2 and 8.9%, while incidence of malignancy in those lesions ranges between 8 and 64% [3]. There are controversial approaches toward FDG-avid thyroid incidentalomas. Some investigators have mentioned that the majority of patients with FDG-avid thyroid incidentaloma have benign lesions and suggest no obligation to perform further evaluation, unless the lesions are clinically suspicious. In contrast, some other studies have concluded that relatively high prevalence of malignancy in these patients mandates additional evaluation to rule out possibility of malignancy. Therefore it is not clear which groups of patients may benefit from further evaluation of incidentally found FDG-avid thyroid lesions [4].

This is a prospective study for evaluation of incidentally found FDG-avid thyroid lesions in patients undergoing FDG PET for an unrelated underlying cancer. After identification of the thyroid incidentaloma, further evaluations including thyroid ultrasound examination, laboratory thyroid function tests, reevaluation of CT component of PET/CT images, and finally ultrasound-guided FNA of FDG-avid lesions were performed.

## 2. Materials and Methods

In this prospective study between September 2016 and February 2017, all cancer patients, referred to PET/CT department of Masih Daneshvari Hospital, were evaluated for presence of FDG-avid thyroid incidentaloma. Among total of 1126 PET/CT studies performed during this time period, 78 patients (7%) were found to have FDG-avid thyroid incidentaloma which were unrelated to their underlying malignancy. The patients who had the history of previous thyroid disease or malignancy were excluded from the study. The remaining patients were requested to read and sign the institutional review board (IRB) approved consent to participate in this study. Finally 18 patients (1.6%) participated in this prospective study and they agreed to perform complementary diagnostic procedures including thyroid ultrasound examination, thyroid function tests, and ultrasound-guided FNA biopsy [5].

For FDG PET/CT examination all cases underwent similar preparing protocol according to the approved guidelines. The patients were fasted for 4–6 hours and blood glucose levels were double-checked before FDG administration to reassure that it is below 150 mg/dl. A focal hypermetabolic thyroid lesion was defined as an area of focally increased FDG uptake corresponding to thyroid gland on the PET/CT images. The presence of calcification within a focal thyroid lesion on the CT images was also considered and documented. To quantify the value of metabolic activity in the FDG-avid thyroid incidentaloma, the maximum rate of Standard Uptake Value (SUV_max_) in that specific lesion was used. Calculation of SUV max value was automatically performed by our ADW 4.5 GE workstation. All PET/CT images were reviewed jointly by an experienced nuclear medicine specialist and radiologist.

After PET/CT procedure and quantification of metabolic activity of thyroid incidentaloma, all patients underwent cervical ultrasound examination by an experienced radiologist and ultrasound characteristics of the incidentaloma including context, echogenicity, and vascularity were evaluated and documented [6].

Subsequently, FNA biopsy of the incidentaloma under the guide of ultrasound was performed and the prepared samples were reviewed by an experienced pathologist, expert in diagnosis of thyroid pathologies. Furthermore, thyroid function tests (TFT) of each patient were also assessed and reported by the laboratory in the same pathology department in Masih Daneshvari Hospital [7].

## 3. Results

Among 1126 PET/CT studies, 78 patients (7%) were found to have FDG-avid thyroid incidentaloma and finally we were able to include 18 patients (1.6%) in our study which is in accordance with similar studies [3]. We had 9 male and 9 female patients. The patient's age ranged from 22 to 76 years old (mean age of 51 years). The underlying malignancies in our patients included two patients with breast ductal carcinoma, two patients with B-cell lymphoma, four patients with colon adenocarcinoma, two patients with lung adenocarcinoma, and one patient with each of the following conditions: great artery vasculitis, synovial sarcoma of the hip, anal canal melanoma, uterine adenocarcinoma, papillary serous carcinoma of the ovary, multiple myeloma, and plantar melanoma and finally one patient with cancer of unknown primary origin.

Quantification of metabolic activity using SUV max index demonstrated a range between 1.4 and 65.4 with mean value of 9.4. Gross specifications of the incidentaloma on CT scan demonstrated 15 patients with noncalcified thyroid lesion. Two patients had microcalcification and one patient had coarse calcification of the thyroid lesion. Also on CT scan, 10 patients had thyroid lesions with specified borders and 8 patients had lesions with unspecified borders.

On ultrasound examination, 15 patients were found to have hypoechoic and three patients had isoechoic thyroid lesions. The thyroid lesions in 15 patients were solid nodules and 3 patients had solid/cystic components. Regarding lesion vascularity, 7 patients had normal vascular patterns and 10 patients had increasing peripheral vascularity. Only one patient had centrally increased vascularity. Laboratory assessment for thyroid function tests demonstrated euthyroid status in all patients.

Finally the results of FNA biopsy in 15 patients were suggestive for benign thyroid lesion and in 3 patients the results were either compatible with or highly suggestive for malignancy. The findings are summarized in Table 1.

**Figure 1:**
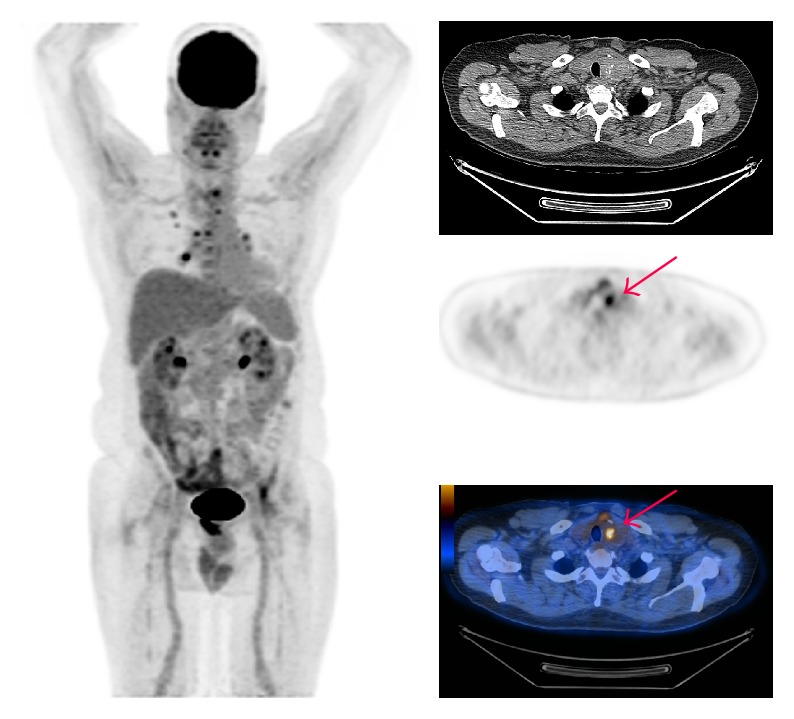
A 63-year-old male with B-cell lymphoma demonstrated bilateral thyroid lobe enlargement with focal increased metabolic activity in left thyroid lobe (Case  8). The arrow only refers to the hypermetabolic incidental thyroid lesion.

**Figure 2:**
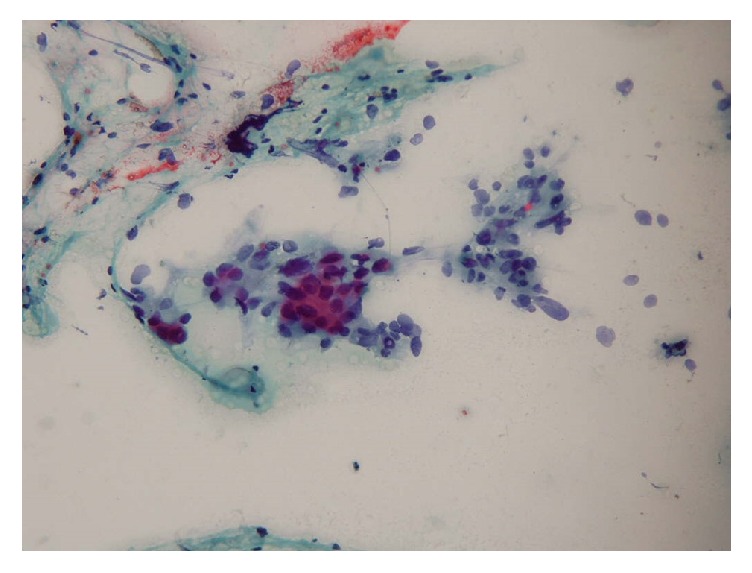
FNA biopsy demonstrates presence of atypical cells as clusters, suggestive for malignancy (Case  8).

**Figure 3:**
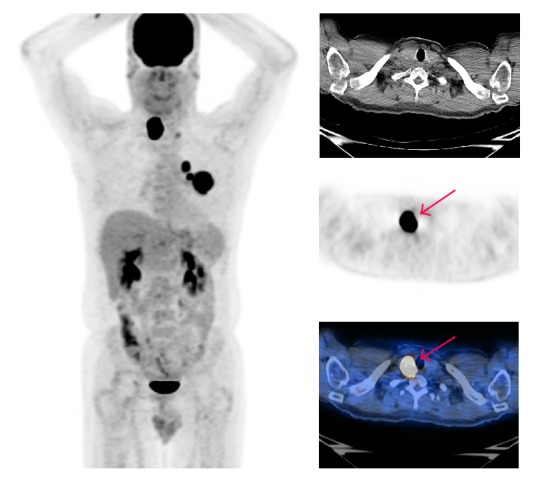
A 56-year-old male with left lung adenocarcinoma demonstrated incidental hypermetabolic thyroid nodule in the right lobe (Case  10). The arrow only refers to the incidental hypermetabolic thyroid lesion.

**Figure 4:**
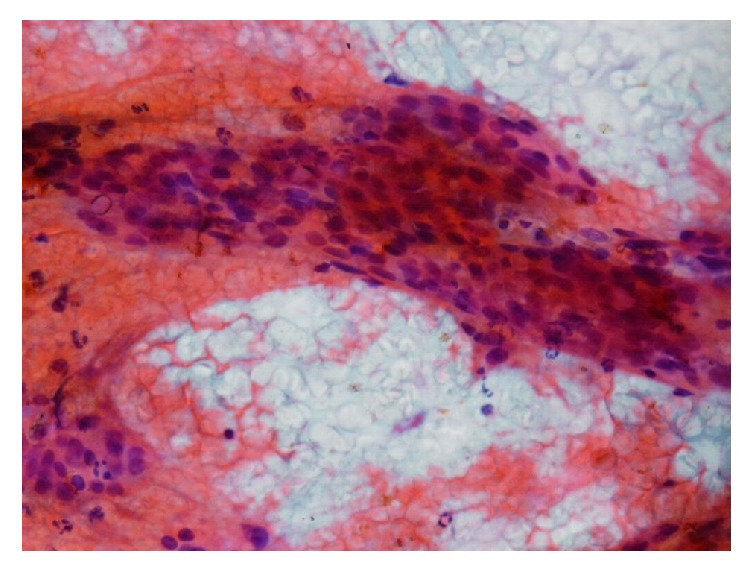
FNA biopsy also demonstrates presence of atypical cells as clusters, highly suggestive for malignancy (Case  10).

The three malignant thyroid incidentaloma cases include A 65-year-old male with primary colon adenocarcinoma (Case  5) with hypermetabolic right thyroid lobe nodule (SUV max = 5.6), A 63-year-old male with primary B-cell lymphoma (Case  8) and diffuse thyroid enlargement with hypermetabolic nodule in left lobe (SUV max = 18.9) and a 56-year-old male with primary lung adenocarcinoma (Case  10) with prominent right thyroid lobe nodule (SUV max = 65.4) (Figures 1–4).

The highest SUV max values were found in patients with lung adenocarcinoma (SUV max = 65.4), B-cell lymphoma (SUV max = 18.9), and synovial sarcoma (SUV max = 14.7) pathology. There was no significant correlation between CT scan and ultrasound characteristics of the lesions and final FNA pathology results. There was significant correlation between SUV max values and FNA final pathology report stating benign versus malignant pathology. In two of the three malignant lesions, the SUV max values were significantly above the calculated average SUV max of 9.4 (Table 1).

## 4. Discussion

We described eighteen cases of FDG-avid thyroid incidentaloma including their PET/CT, ultrasound examination, thyroid function test, and FNA biopsy results.

Many studies have reported usefulness of 18F-FDG PET/CT for distinguishing benign from malignant lesions including thyroid lesions. FDG uptake is usually increased in malignant tissues due to enhanced glycolysis, increased cellular proliferation, and overexpression of glucose transport proteins such as GLUT1. Interestingly the rate of metabolic activity and increased FDG uptake is inversely correlated with degree of tissue differentiation and tissues with less histologic differentiation, usually demonstrating more metabolic activity and FDG uptake on FDG PET imaging [4, 8]. In addition to malignancy, the differential diagnoses of increased FDG uptake in a thyroid lesion include various infectious or inflammatory diseases [8].

The incidence rate (per one year) of thyroid cancer is 2.20 per 100,000 persons in Iran [9]. In our study FNA pathology reports of the three cases (Case  5, Case  8, and Case  10) are either compatible with or highly suggestive for malignancy. Case  8 and case 10 have the highest rate of SUV max among our cases. Case  5 has SUV max equal to 5.6. Although this rate of SUV max is modest among all other cases, however considering the patients with colon adenocarcinoma as the primary tumor, its SUV max level is the highest. This probably emphasizes the importance of performing combined modalities other than 18F-FDG PET/CT in approach to hypermetabolic thyroid incidentaloma. Interestingly in this case (Case  5) other features of the thyroid nodule including unspecified border, presence of calcification, hypoechoic component, and increased vascularity are also in favor of malignancy.

A study by Sasaki et al. mentioned that high FDG uptake in a thyroid lesion is suggestive for malignancy while low levels of SUV max could not entirely rule out the possibility of malignancy [10]. In Brindle et al. investigations, they concluded that malignant lesions are prone to have higher SUV but in some cases, overlap can be noticed [11]. This results are comparable to our patient series.

Ultrasound features of malignant thyroid nodules include any sort of calcifications, irregular and unspecified borders, hypoechoic region in a solid nodule, and presence of unusual or chaotic pattern of vascularity [12]. None of these characteristics are accurate enough to confirm thyroid nodule malignancy and if considered independently, false positive results may increase. In other words, increased number of suspicious ultrasound features is correlated with increased malignancy risk [13]. In our study 15 out of 18 cases had hypoechoic echogenicity and similarly 15 out of 18 patients had solid nodules. If we consider other ultrasound features of thyroid nodule malignancy such as vascularity pattern and nodule border, then fewer patients will meet the malignancy criteria. Seven out of 18 cases in our study including the three malignant cases had unspecified borders. Three cases had thyroid nodule calcifications including 2 of our 3 malignant cases (Case  5 and Case  8). Some studies have demonstrated that calcifications in a thyroid lesion have correlation with malignancy. Shetty et al. confirmed this correlation between ultrasound calcification and malignancy while they said there is no significant correlation between punctuate calcification on CT and malignant or potentially malignant histology in the patients who had biopsy or resection [14].

Regarding noncontrast CT scan findings in FDG-avid thyroid incidentaloma, Kim et al. calculated the ratio of Hounsfield unit (HU) of thyroid incidentaloma compared to normal contralateral thyroid lobe (T/B_HU_). They also calculated the ration of thyroid incidentaloma SUV max compared to liver background. They demonstrated that T/B_HU_ ratio is a simple and effective parameter to stratify the risk of malignancy in thyroid incidentalomas found on PET/CT [15].

Regarding thyroid function test results, all of our patients had euthyroid status. Existence of any correlation between amount of FDG uptake and thyroid function test status has not been widely studied. Pruthi et al. [16] and Karantanis et al. [17] mentioned that there are no significant correlations between SUV level and amount of serum TSH. However, Lee et al. demonstrated a significant relation between the degree of thyroid uptake on 18F-FDG PET/CT and increased serum TSH level [18]. This difference in comments reveals the importance of conducting additional investigations regarding probable correlation between TFT status and level of FDG uptake in thyroid incidentalomas.

Regarding the actual size of FDG-avid thyroid incidentalomas in our study, the largest lesions were found in Cases 2, 5, 8, and 10 (Table 1) where three of them (Cases 5, 8, and 10) had either malignant or highly suspicious thyroid lesions. Likewise, a few other studies have also demonstrated that malignant or potentially malignant nodules are meaningfully larger than benign nodules. Same as other characteristics, there is considerable size overlap between benign and malignant lesions that may limit its role in interpretation of a particular lesion [14].

The major limitation of our study was the small number of participating patients. The patients must have incidentally found hypermetabolic thyroid lesion on a routine 18F-FDG PET/CT study performed for other reasons and they must be willing and also able to participate in our study for further diagnostic investigation including neck ultrasound exam, blood sampling for TFT, and finally FNA biopsy from the thyroid nodule. Because most cancer patients are significantly compromised regarding their physical and emotional health status, the level of participation in such studies is expected to be low as has been encountered in other similar studies. For example, in a study by Hsieh et al. among 477 PET/CT studies they only found 12 hypermetabolic incidentalomas where 10 patients participated for further investigation and finally only 1 malignant case was documented [19]. In a study by King et al. among 15711 PET/CT studies, 22 hypermetabolic incidentalomas were found; all of them participated for further evaluation and finally only 3 cases were diagnosed to be malignant [20]. Interestingly this study has included the largest number of PET/CT studies according to our literature review. Finally the highest number of documented malignant hypermetabolic incidentalomas is described in study by Kang et al. including 12840 PET/CT studies with 1151 incidentalomas, with 190 participating patients and 57 documented malignant incidentalomas [21].

Invasive and debilitating nature of underlying disease in most cancer patients with hypermetabolic thyroid incidentaloma is a significant problem to convince them to participate for further diagnostic investigation. On the other hand, performing long follow-up in these patients is challenging. Further researches with greater patient sample size and longer follow-up are required to better characterize the features of hypermetabolic thyroid incidentalomas.

## 5. Conclusion

Encountering thyroid incidentaloma in 18-F FDG PET/CT scan is not prevalent. Majority of the thyroid incidentalomas are benign lesions and higher rates of SUV max are in favor of malignancy while it should be noticed that low rates of SUV max do not exclude malignancy. Utilizing combined diagnostic modalities in the work-up of hypermetabolic thyroid incidentalomas is helpful to make an accurate decision in patient management. Performing thyroid ultrasound examination and guided FNA biopsy in hypermetabolic thyroid incidentalomas is essential, especially in patients with high rate of FDG uptake.

## Figures and Tables

**Table 1:** General characteristics of the cases.

Case	Age	Gender	Underlying disease	SUV max	TFT^*∗*^	Nature	Echogenicity	Border	Calcification	Vascularity	Size^*∗∗*^	Pathology
(1)	43	F	Breast ductal carcinoma	4.7	Euthyroid	Solid	Hypoechoic	Unspecified	No	Normal	8 × 12	Benign
(2)	51	F	Breast ductal carcinoma	3.5	Euthyroid	Solid	Hypoechoic	Unspecified	No	Normal	6 × 11	Benign
(3)	30	F	Great arteries vasculitis	4.3	Euthyroid	Solid	Hypoechoic	Specified	No	Peripheral increase	5 × 13	Benign
(4)	36	M	Colon adenocarcinoma	3.9	Euthyroid	Solid	Isoechoic	Specified	No	Peripheral increase	14 × 15	Benign
(5)	65	M	Colon adenocarcinoma	5.6	Euthyroid	Solid/cystic	Hypoechoic	Unspecified	Micro	Peripheral increase	25 × 26	Malignant
(6)	61	M	Colon adenocarcinoma	1.4	Euthyroid	Solid	Hypoechoic	Specified	No	Normal	17 × 19	Benign
(7)	54	F	Colon adenocarcinoma	5.1	Euthyroid	Solid	Hypoechoic	Unspecified	No	Normal	8 × 10	Benign
(8)	63	M	B cell lymphoma	18.9	Euthyroid	Solid	Hypoechoic	Unspecified	Micro	Peripheral increase	21 × 30	Malignant
(9)	22	F	Synovial sarcoma	14.7	Euthyroid	Solid	Hypoechoic	Specified	Coarse	Peripheral increase	19 × 22	Benign
(10)	56	M	Lung adenocarcinoma	65.4	Euthyroid	Solid	Hypoechoic	Unspecified	No	Central increase	13 × 45	Malignant
(11)	49	F	Lung adenocarcinoma	5.9	Euthyroid	Solid	Hypoechoic	Unspecified	No	Peripheral increase	7 × 17	Benign
(12)	43	F	Anal melanoma	3.6	Euthyroid	Solid	Isoechoic	Specified	No	Normal	7 × 13	Benign
(13)	32	F	Adenocarcinoma of uterus	8.6	Euthyroid	Solid	Hypoechoic	Specified	No	Peripheral increase	13 × 17	Benign
(14)	72	M	Without any origin	2.7	Euthyroid	Solid/cystic	Hypoechoic	Specified	No	Normal	10 × 12	Benign
(15)	44	F	B cell lymphoma	3.5	Euthyroid	Solid/cystic	Isoechoic	Specified	No	Normal	8 × 10	Benign
(16)	59	F	Papillary serous carcinoma of the ovary	10	Euthyroid	Solid	Hypoechoic	Unspecified	No	Peripheral increase	5 × 10	Benign
(17)	55	M	Multiple myeloma	1.9	Euthyroid	Solid	Hypoechoic	Unspecified	No	Normal	14 × 16	Benign
(18)	62	M	Melanoma of the plantar	5.9	Euthyroid	Solid	Hypoechoic	Unspecified	No	Peripheral increase	7 × 16	Benign

^*∗*^Thyroid function test (values of T3, T4, and TSH are discussed in detail in the results); ^*∗∗*^length*∗*width (mm).
